# Noninvasive fetal genotyping of single nucleotide variants and linkage analysis for prenatal diagnosis of monogenic disorders

**DOI:** 10.1186/s40246-022-00400-4

**Published:** 2022-07-27

**Authors:** Wenman Wu, Xuanyou Zhou, Zhengwen Jiang, Dazhi Zhang, Feng Yu, Lanlan Zhang, Xuefeng Wang, Songchang Chen, Chenming Xu

**Affiliations:** 1 Department of Laboratory Medicine, Ruijin Hospital, Shanghai Jiaotong University School of Medicine, Shanghai, People’s Republic of China; 2 Obstetrics and Gynecology Hospital, Institute of Reproduction and Development, Fudan University, Shanghai, People’s Republic of China; 3 Genesky Diagnostics (Suzhou) Inc., 218 Xinghu St, Suzhou, Jiangsu People’s Republic of China; 4 International Peace Maternity and Child Health Hospital, School of Medicine, Shanghai Jiao Tong University, Shanghai, People’s Republic of China; 5 Shanghai Key Laboratory of Embryo Original Disorders, Shanghai, People’s Republic of China; 6 Collaborative Innovation Center of Hematology, Shanghai Jiaotong University School of Medicine, Shanghai, People’s Republic of China; 7 Shanghai Academy of Experimental Medicine, Shanghai, People’s Republic of China; 8 Research Units of Embryo Original Diseases, Chinese Academy of Medical Sciences (No. 2019RU056), Shanghai, China

**Keywords:** Cell-free DNA, Fetal genotyping, Prenatal diagnosis, Massively parallel sequencing, Monogenic disorder

## Abstract

**Background:**

High-cost, time-consuming and complex processes of several current approaches limit the use of noninvasive prenatal diagnosis (NIPD) for monogenic disorders in clinical application. Thus, a more cost-effective and easily implementable approach is required.

**Methods:**

We established a low-cost and convenient test to noninvasively deduce fetal genotypes of the mutation and single nucleotide polymorphisms (SNPs) loci by means of targeted amplification combined with deep sequencing of maternal genomic and plasma DNA. The sequential probability ratio test was performed to detect the allelic imbalance in maternal plasma. This method can be employed to directly examine familial pathogenic mutations in the fetal genome, as well as infer the inheritance of parental haplotypes through a group of selected SNPs linked to the pathogenic mutation.

**Results:**

The fetal mutations in 17 families with different types of monogenic disorders including hemophilia A, von Willebrand disease type 3, Duchenne muscular dystrophy, hyper-IgM type 1, glutaric acidemia type I, Nagashima-type palmoplantar keratosis, and familial exudative vitreoretinopathy were identified in the study. The mutations included various forms: point mutations, gene inversion, deletions/insertions and duplication. The results of 12 families were verified by sequencing of amniotic fluid samples, the accuracy of the approach in fetal genotyping at the mutation and SNPs loci was 98.85% (172/174 loci), and the no-call rate was 28.98% (71/245 loci). The overall accuracy was 12/12 (100%). Moreover, the approach was successfully applied in plasma samples with a fetal fraction as low as 2.3%.

**Conclusions:**

We have shown in this study that the approach is a cost-effective, less time consuming and accurate method for NIPD of monogenic disorders.

**Supplementary Information:**

The online version contains supplementary material available at 10.1186/s40246-022-00400-4.

## Introduction

Traditional prenatal diagnosis of monogenic disorders relies on fetal DNA obtained via invasive procedures such as amniocentesis and chorionic villous sampling (CVS), which pose a threat to the fetus [1–3]. Since noninvasive prenatal diagnosis (NIPD) has emerged as a popular and reliable detection methods for fetal abnormalities, early detection of monogenic disorders by NIPD is an area of growing interest. The development of NIPD stems from the finding that peripheral blood from a pregnant woman contains fragmented fetal DNA, which constitutes approximately 5–20% of total cell-free DNA (cfDNA) [4, 5]. Cell-free fetal DNA (cffDNA) generally derives from apoptotic trophoblasts in the placenta during pregnancy and clears from the maternal system several hours after giving birth [6–8]. The cffDNA can be first detected in maternal blood as early as 5 weeks of gestation, but the amount of cffDNA at that time is still too low to be tested [9]. In fact, cffDNA usually reaches levels sufficient for prenatal diagnosis of monogenic disorders at around gestation week 6–7 [10]. The discovery of cffDNA in the maternal peripheral blood in combination with the development of next-generation sequencing (NGS) technology has allowed for a wider application of NIPD in clinical practice. Using massive parallel sequencing, researchers can examine copy number and nucleotide sequences by extracting fetal-specific information from a maternal background [11–13].

Initially, NIPD was offered for autosomal dominant disorders of paternal inheritance and de novo conditions, and the detection of achondroplasia is the first step in this clinical implementation [14, 15]. The fetal genotype can be identified by detecting whether the pathogenic mutation of paternal origin is present or not in the maternal plasma. This is called the principle of the absence or presence [16, 17]. In this way, one can perform NIPD for autosomal dominant disorders, de novo conditions and some types of autosomal recessive disorders (parents carrying different mutations) by detecting the foreign alleles in the maternal plasma [18]. But for detection of maternal-origin mutations, the principle of absence or presence is not useful. Given that the maternal plasma cfDNA contains both fetal and maternal DNA fragments, the strong skew toward maternal-derived fragments in the cfDNA is one of the major barriers for deducing the fetal genotype accurately, especially when both parents carry the same pathogenic mutation [10, 19]. For example, in positions where mother is heterozygous and father is homozygous, if the fetal is homozygous for the same position, the inference of fetal genotypes will become complicated and analysis for the allelic imbalance in maternal plasma is required. In the past decades, several approaches and algorithms were investigated for the diagnosis of maternal mutations based on the quantification of allelic imbalance ratio [7, 13, 20–23]. Among them, relative mutation dosage (RMD) and relative haplotype dosage (RHDO) analysis conducted by Lo et al. are the most representative [7, 24]. Using these two dosage-based approaches, the number of reads covering a point mutation or a haplotype is often measured first, then a sequential probability ratio test (SPRT) is used to assess the genetic imbalance, and the over-represented allele or haplotype will be analyzed to determine the fetal genotype. Subsequently, approaches without the need of samples from parents and the affected proband have also emerged [19, 25], and several technologies and algorithms enable the detection of de novo mutations in a precise and sensitive way [13, 26]. However, high-cost, time-consuming and complex processes limit their widespread use in clinical practice.

For any given clinical test, it is essential to assess the accuracy, cost and turnaround time. In this study, we established a widely available and economical approach for NIPD which can accurately infer the fetal genotype for different monogenic disorders. NIFG, which stands for noninvasive fetal genotyping, can be used to diagnose the inheritance of known mutations. We amplified genomic regions (60–120 bp) containing the mutation loci of interest and single nucleotide polymorphisms (SNPs) flanking the mutation loci from maternal genomic and plasma DNA. The amplicons were indexed by a second round of PCR, which were directly subject to paired-end sequencing (150 bp). The paired reads originating from a single molecule were compared for proofreading. The allelic ratios between the maternal genomic DNA and plasma DNA were statistically analyzed to yield fetal genotypes. For each locus, we conducted two parallel experiments and only consistent results were considered.

Using NIFG, pathogenic mutations such as point mutations and small indels can be directly identified. Moreover, linkage analysis can be performed to infer the inheritance of parental haplotypes by genotyping a group of single nucleotide polymorphisms (SNPs) loci adjacent to the causal mutation. This is particularly useful for detection of gene inversion, large indels and repeat expansion, etc. A combined analysis of both the mutation loci and haplotype information can also make up for the shortcomings of the single method and improve the diagnostic accuracy.

## Materials and methods

### Sample collection

Familial mutations in the *CD40LG, DMD, F8, GCDH, SERPINB7, VWF* and *TSPAN12* gene were identified from probands and family members. Whole blood (~ 10 mL) from mother, father, proband and/or the other available family members and amniotic fluid samples (~ 10 mL) were provided for the study. The artificial samples were prepared as follows: 5 mL of whole blood from a female volunteer and her biological child (male) were drawn into EDTA-Vacutainer tubes. All patients were informed of details of the procedure and signed an informed-consent agreement.

Genomic DNAs (gDNAs) were extracted using QIAamp DNA Mini Kit (QIAGEN) and fragmented by Covaris S2 sonicator. DNA concentrations were measured by Qubit3.0 fluorometer (Invitrogen). For NIFG, cfDNA was extracted using QIAamp Circulating Nucleic Acid Kit (QIAGEN), while maternal gDNA was fragmented by Covaris S2 sonicator.

### Sequencing library preparation

Amplification of target genomic regions was carried out in a multiplex PCR (20 μL). The reaction mix contained 1X Q5 High-Fidelity PCR Master Mix (NEB), 500 nM of each primer (Tm 62–65 °C, amplicon length between 60 and 120 bp) and 5 ng of cfDNA/gDNA template. PCR program was as follows: 98 °C for 30 s; 9 cycles of 98 °C for 10 s, 62-0.5 °C per cycle for 30 s, 72 °C for 30 s; 16 cycles of 98 °C for 10 s, 64 °C for 30 s, 72 °C for 30 s. Primer sequences can be found in Additional file 1: Table S1. Both forward and reverse primers had adaptors on the 5’ end that can be bound by index-PCR primers. Adaptor sequences (5′–3′) are: forward primer, ACACTCTTTCCCTACACGACGCTCTTCCGATCT; reverse primer, GACTGGAGTTCAGACGTGTGCTCTTCCGATCT. Products from the first PCR were indexed and purified using Agencourt AMPure XP PCR Purification Kit (Beckman). The program for the index-PCR was: 98 °C for 30 s; 12 cycles of 98 °C for 10 s, 60 °C for 30 s, 72 °C for 30 s. Primer sequences (5′–3′) are: forward, AATGATACGGCGACCACCGAGATCTACACACACTCTTTCCCTACACGACGC and reverse, CAAGCAGAAGACGGCATACGAGATGTGACTGGAGTTCAGACGTGTGCT. Products from the index-PCR were quantified by Qubit dsDNA BR Assay Kit (Invitrogen) and quality-assessed using 2100 Bioanalyzer (Agilent). Purified DNA was diluted to 2 nM in elution buffer (Qiagen). Equal aliquots were pooled to a 2 nM-library and denatured using sodium hydroxide. Final library (10 pM) was mixed with 10 pM PhiX control library for a 15% PhiX spike-in, enhancing sequence diversity. Paired-end 150 bp reads were generated on the Miseq platform (Illumina). Library preparation and sequencing were carried out by Genesky Diagnostics Inc. (Suzhou, China).

### Sequence alignment and depth calculation

Adaptor sequences were trimmed and paired reads were processed as follows: (1) both reads need to have a quality score (*Q*-score) of 30 or higher; (2) the two reads were aligned with unmatched bases marked as “*N*”; (3) paired reads with unambiguous base calling at target site were eligible for analysis, otherwise would be discarded. Filtered reads were normalized and mapped to the human reference genome GRCh37/hg19 using the BWA v.0.7.15 MEM algorithm. The Picard tool was used to convert aligned reads to a binary (BAM) file. The SAMtools v.1.3.1 was used to retrieve the read depth of each base.

### Statistical analysis

Allele counts at each target site in maternal genomic and plasma DNA were compared using SPRT and Chi-squared test. SPRT was conducted according to a previous report [27] with the threshold likelihood ratio set at 1000. Alpha level for Chi-squared test was set at ≤ 0.001 to reject and > 0.01 to accept the null hypothesis. All error bars indicate 95% confidence interval (CI).

## Results

### Rationale of SNP genotyping and detection of allelic imbalance

**Fig. 1:**
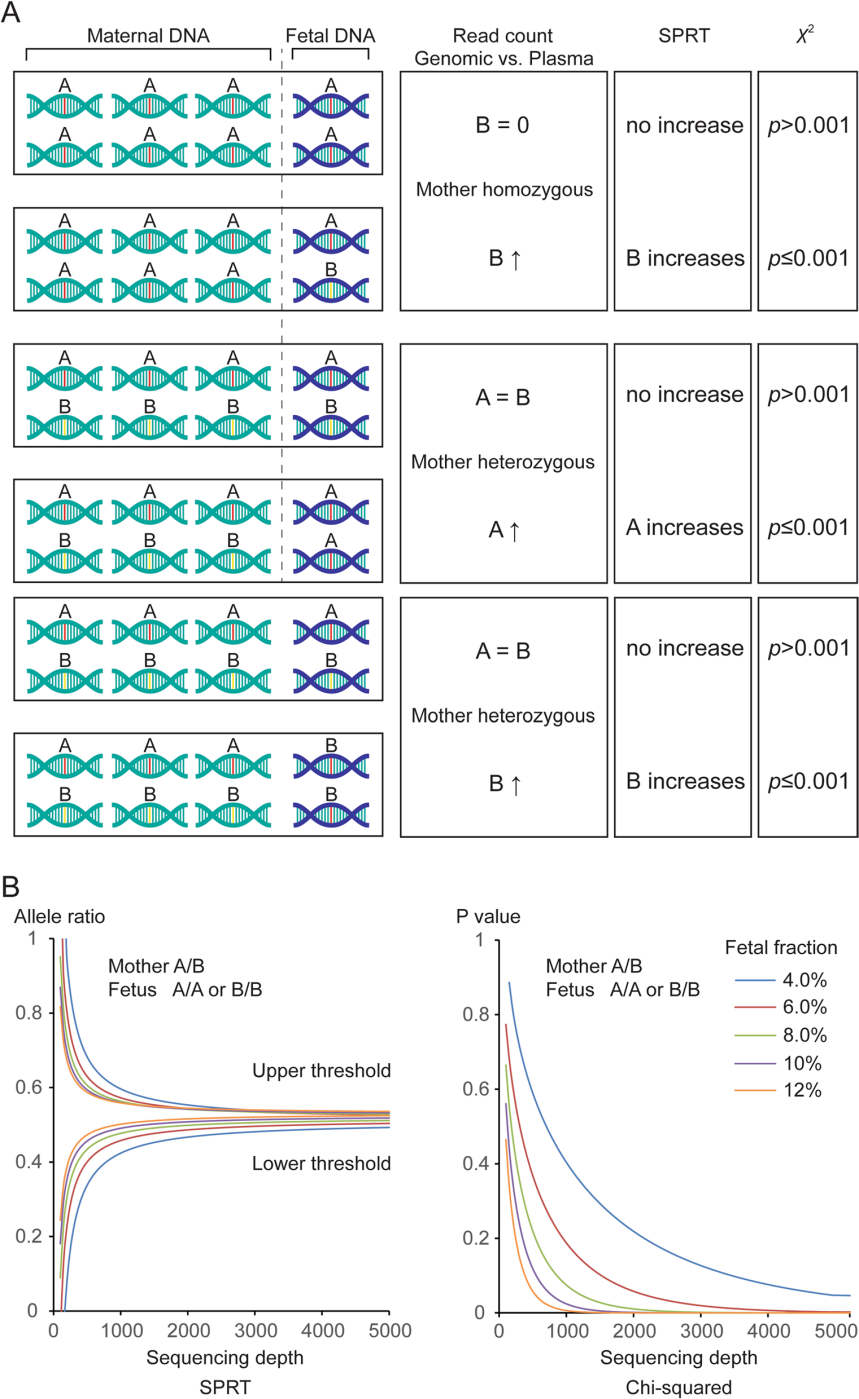
The rationale of NIFG in fetal genotyping. **A** The illustration showing how the allelic imbalance between maternal genomic DNA and plasma DNA can be used to infer the genotype of the fetus. **B** Simulated SPRT and Chi-squared test results when mother is heterozygous and fetus is homozygous for a given SNP locus

Without knowing the paternal genotype, if the mother is homozygous (A/A) for a SNP locus, the fetus could be A/A or A/B, in which “A” and “B” represent the two possible alleles wild or mutant. If mother is heterozygous (A/B), then the fetus could be A/A, B/B or A/B. When fetal and maternal genotypes differ, the paternal allele will be over-represented in plasma DNA (contains fetal DNA) relative to maternal DNA (does not contain fetal DNA). The allelic imbalance can be detected via amplicon sequencing and comparison of the allele count. Alternatively, if fetal and maternal genotypes are identical, no such imbalance will be present (Fig. 1A).

The degree of the allelic increase depends on fetal fraction and the detection sensitivity is determined by sequencing depth and error rate. We evaluated two statistical methods for their performance in detecting allelic imbalance: sequential probability ratio test (SPRT) and Pearson's Chi-squared test (*χ*^2^).

SPRT allows two hypotheses to be compared as data accumulate, which has been successfully applied to detect allelic imbalance in the diagnosis of monogenic disorders by NIPD [7, 24]. On the other hand, Chi-squared test examines whether an observed allele frequency distribution deviates from an expected value. In both scenarios, the null hypothesis is that fetal and maternal genotypes are identical. When mother is homozygous (AA or BB) with the fetus being heterozygous (AB), the presence of the paternal allele in plasma DNA would be recognized at low sequencing depth since it is absent from the maternal genome. In contrast, when mother is heterozygous (AB) and the fetus is homozygous (AA or BB), both alleles are present in the maternal sample, making it more challenging to detect the over-represented maternal allele.

In Fig. 1B, we simulated the results of two statistical tests performed at maternal heterozygous, fetal homozygous SNPs. We assumed an equal distribution of read counts between the two alleles (no amplification bias). We used a likelihood threshold of 1000 for SPRT and set the alpha level at 0.001 for the Chi-squared test. As expected, the sequencing depth required to reach statistical significance was inversely correlated with fetal fraction. For instance, in SPRT, 8650X and 1000X yielded correct fetal genotypes at 4% and 12% fetal fraction, respectively. For Chi-squared test, the same outcome was achieved at 13400X and 1450X, respectively.

### The accuracy of SNP genotyping

In a proof-of-concept study, we selected 36 SNPs (minor allele frequency, MAF > 0.3, 1000 genomes) from all chromosomes in the human genome except for 13, 17 and Y. These loci included single-nucleotide changes as well as indels of 2–5 nucleotides (Additional file 2: Table S2). We prepared artificial samples in which genomic DNA from a female volunteer and her biological child were fragmented by ultrasonication and mixed at pre-determined proportions to represent plasma DNA with a fetal fraction of 4%, 6% and 8%. In addition, we collected genomic and plasma DNA from five pregnancies. Fetal fraction was estimated by calculating the percentage of paternal alleles at maternal homozygous and fetal heterozygous SNPs [28]. The amniotic fluid samples (~ 10 mL) were also obtained for verification of the NIFG results.

**Fig. 2:**
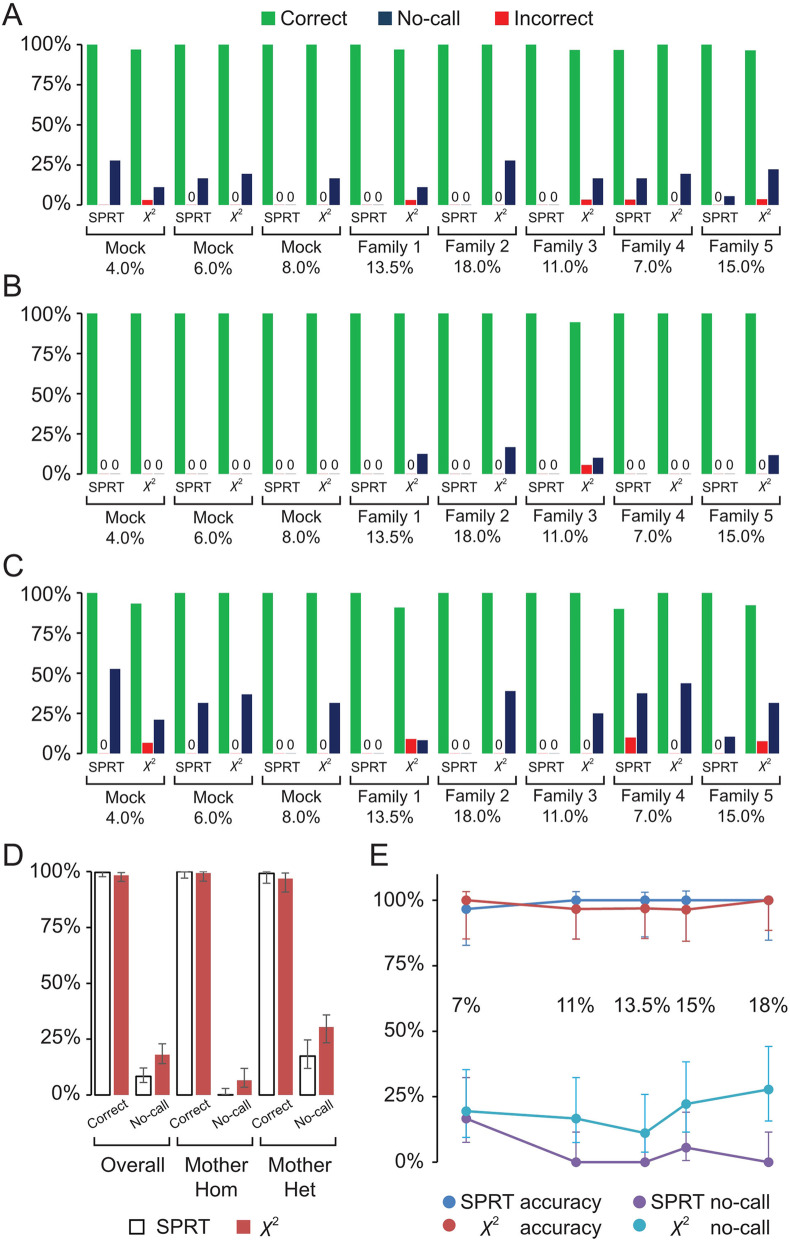
The performance of SPRT and Chi-squared test in detecting allelic imbalance. Bar graphs showing the percentage of correct, incorrect and no-call genotyping results in **A** 36 SNPs, **B** maternal homozygous SNPs, **C** maternal heterozygous SNPs across all samples tested. **D** Bar graph indicating the overall accuracy of SPRT and Chi-squared test in fetal genotyping. **E** Scatter plot displaying the accuracy and no-call rate of SPRT and Chi-squared test as fetal fraction increases. All error bars indicate 95% CI

Thirty-six SNPs were amplified from 5 ng of genomic and plasma DNA in a multiplex reaction. A subsequent index-PCR was performed to add sequencing adaptors on both ends of amplicons, which were subject to massively parallel sequencing on the Miseq platform (150 bp paired-end, average depth: 15500X). The experiments were repeated twice, and only concordant genotyping results were considered; otherwise, a “no-call” was assigned. Overall, SPRT yielded 99.62% (95% CI 97.67–99.99%) accuracy in base calling (8.33% no-call), compared with 98.31% (95% CI 95.57–99.50%) for Chi-squared test (18.06% no-call) (Fig. 2A, D). For maternal homozygous SNPs, SPRT was 100% (95% CI 97.00–100.00%) accurate (zero no-call), while Chi-squared test was 99.29% (95% CI 95.66–99.99%) with 6.67% no-call (Fig. 2B, D). For maternal heterozygous SNPs, SPRT results were 99.12% (95% CI 94.71–99.99%) correct (17.39% no-call), compared with 96.88% (95% CI 90.83–99.32%) for Chi-squared test (30.43% no-call) (Fig. 2C, D).

Fetal fractions ranged from 7 to 18% in five plasma samples. The accuracy of two statistical tests in fetal genotyping remained consistent at different fetal fractions. However, the no-call rate was inversely correlated with fetal fraction, especially for SPRT (Fig. 2E). The correlation coefficient was negative 0.72 (− 0.72) for SPRT and 0.47 for Chi-squared test. Notably, Chi-squared test resulted in significantly higher no-call rate when compared to SPRT (Fig. 2E). Thus, we elected to use SPRT in subsequent experiments.

### Haplotype construction and linkage analysis

We have aforementioned that NIFG is capable of detecting mutations including single nucleotide changes and small indels. However, in order to improve the diagnostic accuracy as well as detect other types of complex mutations such as inversion, repeat expansion and large indels, haplotype construction and linkage analysis were also performed to infer the inheritance pattern. This is achieved by: (1) selecting a group of SNPs that immediately flank the mutation (preferably within 200–500 kb); (2) genotyping these SNPs in the proband, parents, fetus therefore the affected haplotypes can be identified.

**Fig. 3:**
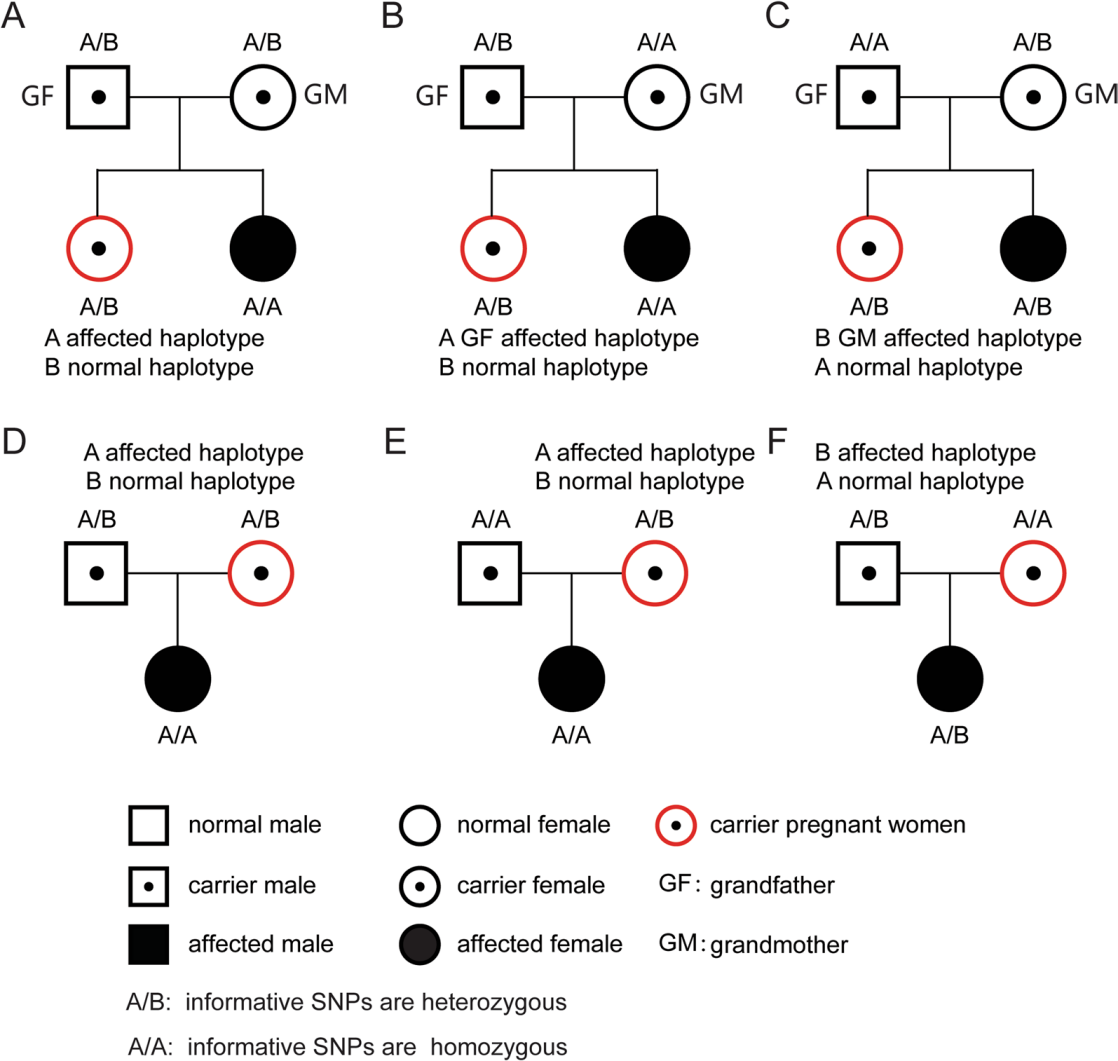
Haplotype and linkage analysis for families with autosomal recessive disorders. Hypothetical families in which **A**, **B**, **C** a sibling of the pregnant woman is a patient and **D**, **E**, **F** a previously born child is a patient. In both cases, the pregnant women are heterozygous carriers of the pathogenic mutation. Different conditions show how the haplotype and linkage analysis can be used to identify the affected haplotype linked to the pathogenic mutation. **A** Both grandparents are heterozygous for selected SNPs; **B** GM is homozygous and GF is heterozygous for selected SNPs; **C** GF is homozygous and GM is heterozygous for selected SNPs; **D** Both parents are heterozygous for selected SNPs; **E** the mother is heterozygous and the father is homozygous for selected SNPs; **F** the father is heterozygous and the mother is homozygous for selected SNPs

In fact, to detect maternally inherited variants, we analyzed SNPs that were heterozygous in mother and homozygous in father, while for detection of paternally inherited variants, selected SNPs were heterozygous in father and homozygous in mother as described by Lo et al.[7]. Taking autosomal recessive disorders as an example, two possible scenarios are considered: first, a sibling of the parent is a patient (Fig. 3A, B, C); and second, a previously born child is a patient (Fig. 3D, E, F). In the first scenario, in order to determine the affected maternal haplotype and whether it is inherited from grandfather (GF) or grandmother (GM), one of the following conditions needs to be met: (1) selected SNPs were homozygous in one grandparent and heterozygous in the other: a. SNPs homozygous in GM and heterozygous in GF were used to determine whether the affected haplotype is inherited from GF (Fig. 3B); b. SNPs heterozygous in GM and homozygous in GF were used to determine whether the affected haplotype is inherited from GM (Fig. 3C); (2) both grandparents are heterozygous for selected SNPs, and the genotype of the proband at the said SNP differs from at least one of the parents (Fig. 3A). In the second scenario, in order to determine the affected haplotype in both parents, one of the following conditions needs to be met: (1) selected SNPs were homozygous in one parent and heterozygous in the other: a. SNPs heterozygous in mother and homozygous in father are used to determine which maternal haplotype is affected (Fig. 3E); b. SNPs homozygous in mother and heterozygous in father are used to determine which paternal haplotype is affected (Fig. 3F); (2) both parents are heterozygous for selected SNPs and the genotype of the proband at the said SNP differs from at least one of the parents (Fig. 3D).

### Diagnosis of inherited monogenic disorders

**Fig. 4:**
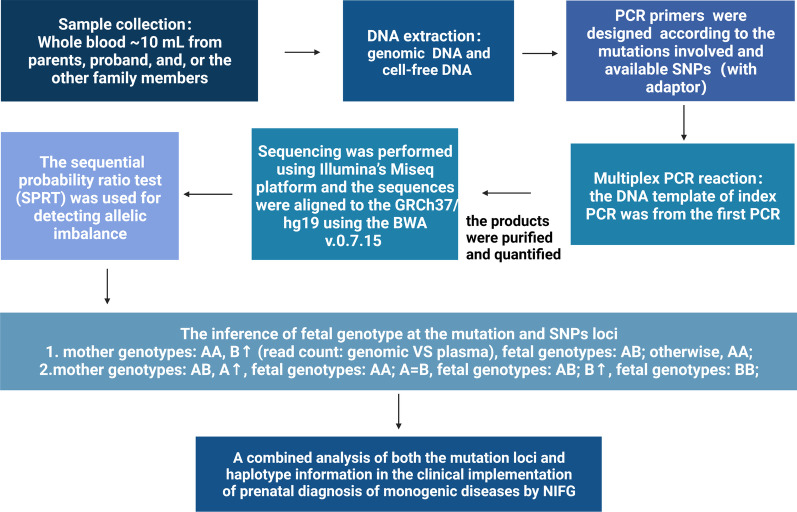
The workflow for NIFG

**Fig. 5:**
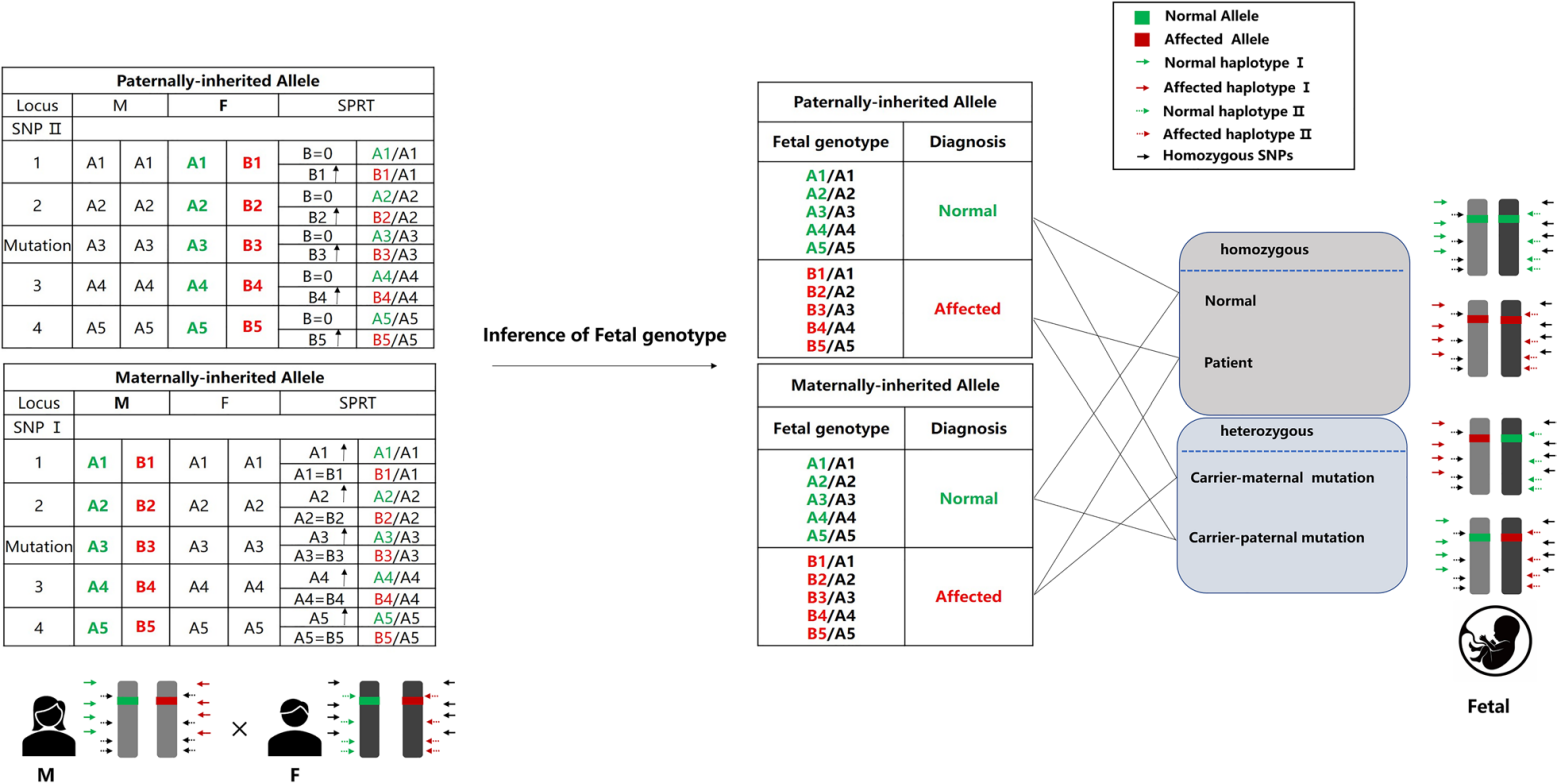
A schematic for the diagnosis of autosomal recessive disorders by NIFG. Parental Haplotypes were firstly constructed accordingly to the selected SNPs linked to the mutation. SPRT statistical analysis was then performed to detect allelic imbalance. Based on all the information above, the fetal genotypes could be deduced. SNP I: to detect maternally inherited variants, selected SNPs were heterozygous in mother **(**A/B**)** and homozygous in father**(**A/A**)**;SNP II: to detect paternally inherited variants, selected SNPs were heterozygous in father **(**A/B**)** and homozygous in mother **(**A/A**)**

The overall workflow diagram is shown in Fig. 4. NIFG is a haplotype-based method also focusing on the mutation loci, which was performed using targeted amplification combined with deep sequencing of maternal genomic and plasma DNA. The allelic ratios between the maternal genomic and plasma DNA were then analyzed by SPRT to detect allelic imbalance. A combined analysis of both the mutation loci and haplotype information was used in the imputation of fetal genotype and clinical diagnosis of monogenic disorders. For instance, the illustration for the diagnosis process of autosomal recessive disorders by NIFG is shown in Fig. 5. The selected SNPs used in the analysis can be divided into two categories including SNP I and SNP II. SNP I was defined as a group of SNPs where mother is heterozygous and father is homozygous, and SNP II was defined as a group of SNPs where mother is homozygous and father is heterozygous. The fetal genotypes could be deduced based on the results of genotyping at the mutation loci and SNPs adjacent to the mutation loci.

In the study, NIFG was used to analyze the parental inheritance of the fetus in 17 families with different monogenic disorders, including X-linked recessive disorders (Hemophilia A, Duchenne muscular dystrophy, hyper-IgM type 1), autosomal recessive disorders (glutaric acidemia type I, Nagashima-type palmoplantar keratosis and Von Willebrand disease type 3) and autosomal dominant disorder (Familial exudative vitreoretinopathy).

### Diagnosis of X-linked recessive disorders by NIFG

NIFG was performed for twelve families with X-linked recessive disorders and made up 70.6% of all NIFG for monogenic disorders, including ten cases of Hemophilia A, one case of Duchenne muscular dystrophy (DMD) and one of hyper-IgM type 1 (HIGM1).

Hemophilia A is an X-linked, recessive disorder due to deficiency of factor VIII (encoded by the *F8* gene), which is critical for blood clotting. Mutations in F8 that eliminate or reduce its expression result in severe, moderate and mild hemophilia, respectively [29]. The incidence of hemophilia A is 1/5000 in male live births, and ~ 70% of cases are inherited [29]. In this study, we performed NIFG on DNA samples of ten pregnant women who carried *F8* mutations, including point mutations, deletion, duplication and intron 22 inversion. In addition to mutation sites, we designed primers for 19 single nucleotide polymorphisms (SNPs) (MAF > 0.02, linkage disequilibrium *r*2 < 0.8) that were selected from a region encompassing *F8* that spans ~ 1.7 Mb on chromosome X (Additional file 3: Table S3). These loci were sanger-sequenced in the mother, proband and maternal grandparents (if proband is not available). Amelogenin loci were also amplified from plasma DNA and sequenced to determine fetal gender. We performed two parallel experiments and only considered concordant results (otherwise no call). Further, linkage analysis was performed to infer the inheritance of parental haplotypes by analyzing a group of selected SNPs loci (as described above). Based on all the information above, the fetal genotypes could be deduced. We successfully identified 3 male patients, 1 female carrier and 6 normal fetuses from 10 pregnancies (Table 1, Additional file 4: Table S4), and the results were confirmed by sequencing of amniotic fluid samples. The average sequencing depth per locus in the *F8* gene was 15339X.

**Table 1:**
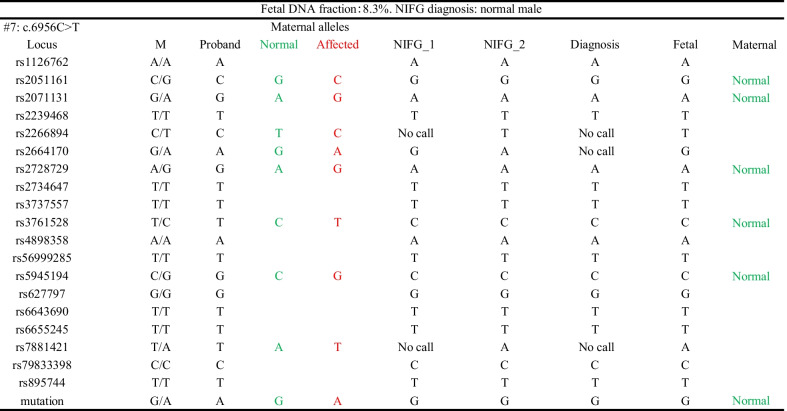
NIFG results for diagnosis of hemophilia A

Affected alleles are shown in red, while the normal alleles are in green. *M* mother, Fetal: genotyping results obtained from amniotic fluid

The same processes of prenatal analysis using NIFG were also performed on the other families with X-linked disorders. In the DMD family, we detected a TCTA 4-base pair (bp) deletions/insertions (delins) located in the exon 8 of *DMD* gene in the proband and his mother was a carrier, and the results of NIFG revealed that the fetus was a female carrier of the mutation (Additional file 4: Table S4). In the HIGM type 1 family, the mutation in the proband was inherited from the mother carrying a missense mutation of *CD40LG* (c.676G > A, p. Gly226Arg); unfortunately, we found that the fetus was also a male patient of the point mutation (Additional file 4: Table S4). Selected SNPs of both genes are summarized in Additional file 3: Table S3. The average sequencing depth per locus was 18693X in the *DMD* gene and 42545X in the *CD40LG* gene. Notably, NIFG for X-linked recessive disorders provided correct diagnosis when fetal fraction was as low as 2.30%.

### Diagnosis of autosomal recessive disorders by NIFG

Four families with three autosomal recessive disorders including one family of glutaric acidemia type I (GA-1), one family of Nagashima-type palmoplantar keratosis (NPPK) and two families of Von Willebrand disease (VWD) type 3 were recruited and analyzed.

Von Willebrand factor, encoded by the *VWF* gene, is a plasma protein that contributes to the formation of a platelet thrombus and protects FVIII from degradation via binding. Deficiency or structural defects of VWF lead to Von Willebrand disease (VWD) [30]. Our study recruited two VWD type 3 families, which is the most severe form of this autosomal recessive disorder (complete absence of VWF protein) [31]. Genetic evaluation of the proband revealed a homozygous intronic mutation (c.2547-13 T > A) in the first family, indicative of consanguineous marriage. This mutation activated a cryptic splice acceptor and caused an inclusion of 37 bp of intron 19 in the mRNA, resulting in a premature stop codon (p.Cys849Trpfs*28). For another family, we detected two heterozygous *VWF* mutations in the probands (c.7822C > T and c.7403G > C). In addition to the point mutations, we selected 25 SNPs (MAF > 0.2, linkage disequilibrium *r*^*2*^ ≤ 0.61) from a 0.88 Mb region that contains *VWF* gene (Additional file 3: Table S3). As shown in Table 2 and Additional file 5: Table S5, for all families, the fetus was a carrier and only the paternal mutation was detected in the fetus. The conclusions were drawn from fetal genotypes of the mutation loci and further corroborated by multiple SNPs in the linkage analysis.

**Table 2:**
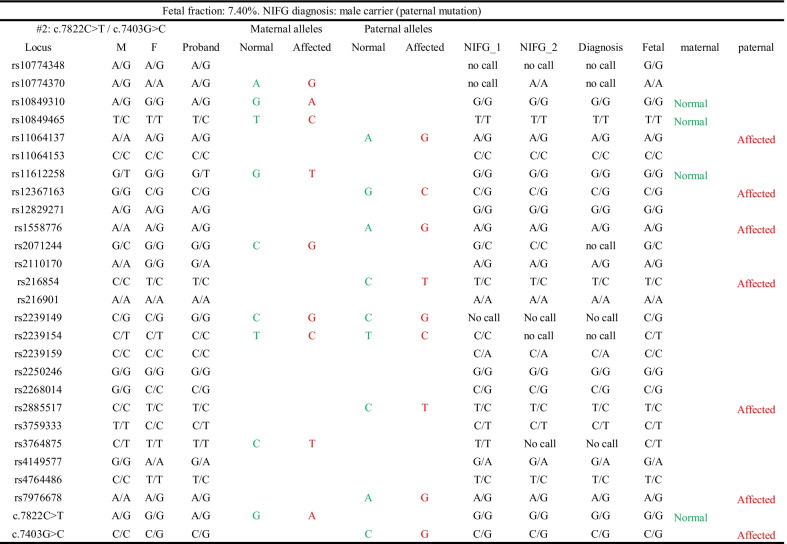
NIFG results for diagnosis of Von Willebrand disease type 3

Affected alleles are shown in red, while the normal alleles are in green. *M* mother, *F* father, Fetal: genotyping results obtained from amniotic fluid

Glutaric acidemia type I (GA-1, OMIM 231,670), an autosomal recessive neurometabolic disorder caused by biallelic pathogenic variants in *GCDH* resulting in deficiency of glutaryl-CoA dehydrogenase (GCDH), is one of the most common inherited metabolic disorders. Approximately 1 in 100,000 children in the world suffers from this disease [32]. In the affected family, the couple were both carriers of the pathogenic variants in the *GCDH* gene (c.416C > G and c.1244-2A > C) and had already given birth to a child diagnosed with GA-1. We diagnosed the fetus as a carrier of the paternal mutation (Additional file 5: Table S5). For the Nagashima-type palmoplantar keratosis (NPPK) family, the wife was a patient of NPPK carrying a homozygous disease-causing variant in the *SERPINB7* gene (c.522dupT), and we detected a heterozygous pathogenic *SERPINB7* mutation in her husband (c.796C > T). In order to have a healthy child, the couple attended to our center for prenatal diagnosis. However, the fetus was diagnosed as a patient by NIFG (Additional file 5: Table S5). Moreover, NIFG for autosomal recessive disorders provided correct diagnosis when fetal fraction was in the range of 4.2–13.5%.

### Diagnosis of autosomal dominant disorders by NIFG

Familial exudative vitreoretinopathy (FEVR, OMIM 133,780) is a complex genetic disorder characterized by incomplete development of the retinal blood vessel [33], which serve as one of the main causes of retinal detachment and eye blindness in adolescents [34]. In this study, we recruited members of one affected family which was clinically diagnosed with FEVR type 5 (FEVR5), and a missense heterozygous mutation of the *TSPAN12* gene (c.566G > A, p.Cys189Tyr) was detected in the mother. The family pedigree indicated that the case was inherited from an autosomal dominant (AD) manner. We designed primers for the point mutation and 29 SNPs (MAF > 0.2, linkage disequilibrium *r*^*2*^ ≤ 0.8) from a 1.7 Mb region that contains *TSPAN12* gene, as shown in Additional file 3: Table S3. By analyzing the results of genotyping at the mutation and linked SNPs loci by NIFG, we identified the fetus as a patient of FEVR5 (Table 3). The fetal fraction detected in this case was 7.3%.

**Table 3:**
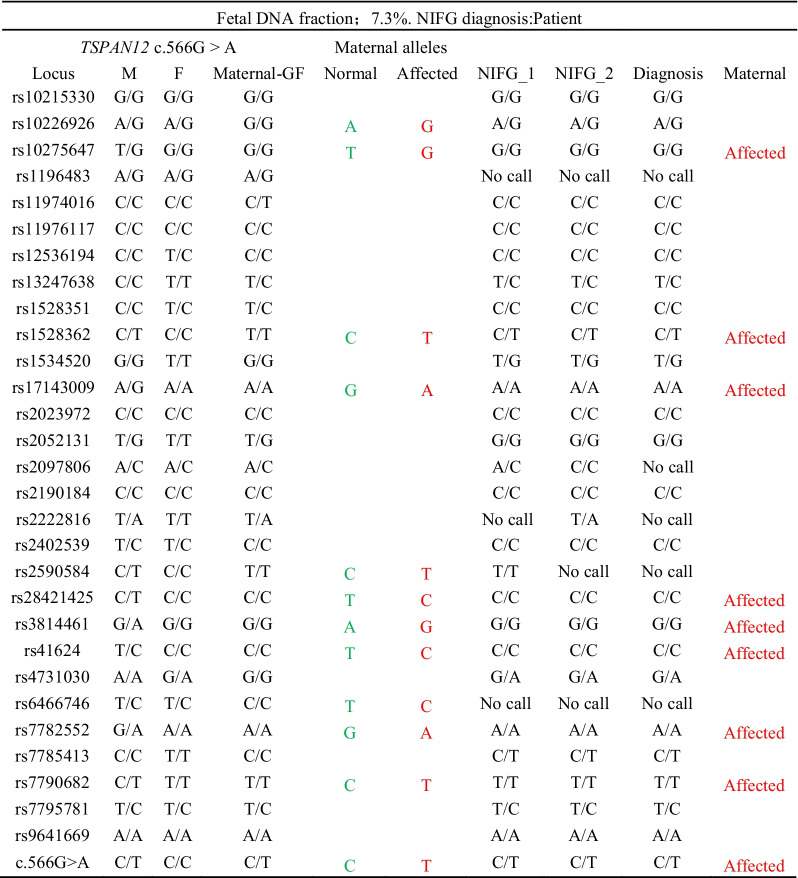
NIFG results for diagnosis of FEVR type 5

Affected alleles are shown in red, while the normal alleles are in green. *GF* grandfather, *M* mother, *F* father

## Discussion

### Cost-effectiveness of NIFG

At present, relative haplotype dosage (RHDO) analysis is served as a common haplotype-based method for the inference of the fetal genotype and diagnosis of certain monogenic disorders. Genome-wide NIPD of monogenic disorders based on RHDO or haplotype was generally performed using whole-genome sequencing (WGS) and whole exome sequencing (WES) and nearly millions of SNPs were required in the analysis [7, 22]. However, to achieve WGS/WES, higher proportion of fetal cfDNA, deeper sequencing depth and coverage will be needed to accurately deduce the parental inheritance of fetus. Targeted sequencing of relevant genomic regions has become increasingly widespread in clinics because of possessing the advantages of low cost and high efficiency. Targeted RHDO analysis has been previously reported to detect parental mutations in β-thalassemia, hemophilia, congenital adrenal hyperplasia (CAH), Ellis-van Creveld syndrome (EVC), and Hunter syndrome [21, 25, 35]. NIFG is a cost-effective, less time consuming and accurate method to noninvasively deduce fetal genotypes by means of targeted amplification combined with deep sequencing. Without the need for typical library preparation steps and complex experimental design, the workflow could be completed within 5 days, offering clinicians the opportunity for fast diagnosis of multiple monogenic disorders. The approach is less expensive and less labor-intensive because WGS/WES technologies were not required. The major advantage of PCR-based amplification is high flexibility, and the approach is therefore applicable for different pathogenic mutations in multiple genes. Additionally, read length exceeded the size of PCR amplicons and can be used to proofread in order to minimize errors. The comparison between the NIFG and RHDO methods is shown in Additional file 6: Fig. S1.

In all 17 families, 12 families were verified by the sequencing results of amniotic fluid samples, the accuracy of NIFG in fetal genotyping at the mutation and SNPs loci was 98.85% (172/174 loci), and the no-call rate was 28.98% (71/245 loci). The overall accuracy of NIFG was 12/12 (100%). The no-calls were partly due to the experimental design that two replicates were performed for each site and only consistent results were considered. In addition, PCR bias could lead to no-calls, since differences in amplification efficiency between two alleles would skew the allele count. This was observed as the ratio of multiple maternal heterozygous SNPs deviated from 1:1 (Additional file 7: Table S6). In this study, several steps were taken to alleviate this problem: (1) the amplicon was restricted to 60–120 bp to accommodate the fragment size of plasma DNA; (2) equal amounts of maternal genomic and plasma DNA were used; (3) stringent threshold was used in the statistical test; (4) more than 19 SNPs/gene were tested, and only one was necessary for diagnosis.

### Clinical implementation of NIFG

NIFG provided diagnoses of monogenic disorders in pregnancies with a fetal fraction ranging from 2.3 to 13.5%, especially for plasma samples with low fetal fractions. For example, In Hemophilia A family #5, NIFG generated 16 correct genotypes (16/16) and 3 no-calls (3/19) when the fetal fraction was 3.09% (Additional file 4: Table S4). NIFG predicted 26 correct genotypes (26/26) and 10 no-calls (10/36) in the artificial sample containing 4% fetal DNA (Fig. 2A). The high-sensitivity of NIFG indicates that it could be used in early pregnancies when fetal fractions are generally low.

Additionally, for the NPPK family, a puzzling result was observed in the detection for the disease-causing variant c.522dupT of maternal origin. The results of NIFG based on informative SNPs showed that the fetal is a patient of NPPK, while we observed a wrong imputation in the mutation loci. The fetal should carry a heterozygous SERPINB7 mutation of c.522dupT, whereas the result of NIFG showed the fetal carry a wild-type genotype (Additional file 5: Table S5). Finally, invasive prenatal diagnosis corroborated the results of SNPs showing the fetal is a patient of NPPK carrying a heterozygous SERPINB7 mutation of c.[522dupT]; [796C > T]. The same situation occurred in the DMD family, the mutant allele (c.826_831 + 1delinsTCTA) was not detected to be present in the maternal plasma (Additional file 4: Table S4). The fetus was finally diagnosed as a female carrier of the mutation via linkage analysis, and the result was confirmed by invasive prenatal diagnosis. The puzzling results may be because the levels of fetal cfDNA in maternal circulation are relatively low and cfDNA usually exist in fragmented forms. The short length of the cffDNA usually makes the testing of deletions, duplications and rearrangements challenging [10]. Our study demonstrated that the results of NIPD used for prenatal diagnosis of monogenic disorders can be unreliable if only the mutation loci are analyzed. Thus, we recommend a combined analysis of both the mutation loci and linkage analysis in the clinical implementation of prenatal diagnosis of monogenic disorders by NIPD.

For future preclinical and clinical applications of NIFG, we propose the following standards to further guarantee accuracy: (1) For point mutations and small indels, both direct genotyping of the mutation and SNP-based linkage analysis are taken into consideration. A minimum of two informative SNPs are required with one located upstream and the other downstream of the mutation. Moreover, there should be no SNP within 500 kb flanking regions of the mutation that contradicts the diagnosis; otherwise, a re-test/re-draw is needed; (2) for mutations that cannot be directly detected by NIFG, only SNP-based linkage analysis is available. Therefore, a minimum of four informative SNPs are mandatory, with at least two SNPs positioned upstream and downstream of the mutation. Similarly, conflicting results from SNPs within 500 kb flanking regions of the mutation are not allowed. In rare occasions where NIFG fails to yield informative results after a re-test, we recommend invasive procedures rather than repeating the assay.

To avoid the condition when mother was homozygous for all SNPs, making the linkage analysis impossible to perform, we recommend a panel of more than 20 SNPs. These loci should be highly polymorphic with low pairwise linkage disequilibrium (preferably MAF > 0.3 and *r*^*2*^ < 0.8) and chosen from a region as small as possible to ensure genetic linkage with the mutation (preferably 200–500 kb flanking regions).

### Limitations of NIFG and possible solutions

NIFG is intended for the diagnosis of known inherited monogenic disorders. But for detection of de novo mutations, the approach is not applicable and whole-genome sequencing method is required. Primers used in NIFG are designed on a case-by-case basis according to the mutations involved and available SNPs. However, pre-made panels targeting genes that are known for causing disorders can be prepared in advance. Moreover, except for point mutations and small indels, NIFG cannot directly detect gene inversion, large indels etc. For these types of pathogenic mutations, linkage analysis can be performed.

## Conclusions

In summary, we established a cost-effective, feasible and convenient haplotype-based approach to infer the fetal genotype for monogenic disorders. A wide spectrum of monogenic disorders including X-linked recessive disorders, autosomal dominant disorders and autosomal recessive disorders can be reliably diagnosed via this approach.

## Supplementary Information

**Additional file 1.** **Table S1**: Primer sequences for multiplex amplification of SNV loci.

**Additional file 2.** **Table S2**: Information of 36 SNPs for evaluation of NIFG.

**Additional file 3.** **Table S3**: SNPs selected for linkage analysis of each gene.

**Additional file 4.** **Table S4**: NIFG results for diagnosis of X-linked recessive disorders.

**Additional file 5.** **Table S5**: NIFG results for diagnosis of autosomal recessive disorders.

**Additional file 6**. **Figure S1**: The similarities and differences between NIFG and RHDO methods.

**Additional file 7.** **Table S6**: Allele depth for all samples used in NIFG.

## Data Availability

The original contributions presented in the study are included in the article/Supplementary Material.

## Abbreviations

CVS: Chorionic villous sampling
NIPD: Noninvasive prenatal diagnosis
cfDNA: Cell-free DNA
cffDNA: Cell-free fetal DNA
NIPT: Noninvasive prenatal testing
RMD: Relative mutation dosage
RHDO: Relative haplotype dosage
SPRT: Sequential probability ratio test
NIFG: Noninvasive fetal genotyping;
SNP: Single nucleotide polymorphism
DMD: Duchenne muscular dystrophy
HIGM1: Hyper-IgM type 1
VWD: Von Willebrand disease
NPPK: Nagashima-type palmoplantar keratosis
GA-1: Glutaric acidemia type I
GCDH: Glutaryl-CoA dehydrogenase
FEVR: Familial exudative vitreoretinopathy
WGS: Whole-genome sequencing
WES: Whole exome sequencing

## References

[1] Tabor A, Philip J, Madsen M, Bang J, Obel EB, Nørgaard-Pedersen B (1986). Randomised controlled trial of genetic amniocentesis in 4606 low-risk women. Lancet.

[2] Tabor A, Alfirevic Z (2010). Update on procedure-related risks for prenatal diagnosis techniques. Fetal Diagn Ther.

[3] Kong CW, Leung TN, Leung TY, Chan LW, Sahota DS, Fung TY (2006). Risk factors for procedure-related fetal losses after mid-trimester genetic amniocentesis. Prenat Diagn.

[4] Lo YM, Corbetta N, Chamberlain PF, Rai V, Sargent IL, Redman CW (1997). Presence of fetal DNA in maternal plasma and serum. Lancet.

[5] Vossaert L, Wang Q, Salman R, McCombs AK, Patel V, Qu C (2019). Validation studies for single circulating trophoblast genetic testing as a form of noninvasive prenatal diagnosis. Am J Hum Genet.

[6] Lun FMF, Chiu RWK, Chan KCA, Leung TY, Lau TK, Lo YMD (2008). Microfluidics digital PCR reveals a higher than expected fraction of fetal DNA in maternal plasma. Clin Chem.

[7] Lo YMD, Chan KCA, Sun H, Chen EZ, Jiang P, Lun FMF (2010). Maternal plasma DNA sequencing reveals the genome-wide genetic and mutational profile of the fetus. Sci Transl Med.

[8] Prefumo F, Paolini D, Speranza G, Palmisano M, Dionisi M, Camurri L (2019). The contingent use of cell-free fetal DNA for prenatal screening of trisomies 21, 18, 13 in pregnant women within a national health service: a budget impact analysis. PLoS ONE.

[9] Lo YM, Tein MS, Lau TK, Haines CJ, Leung TN, Poon PM (1998). Quantitative analysis of fetal DNA in maternal plasma and serum: implications for noninvasive prenatal diagnosis. Am J Hum Genet.

[10] Scotchman E, Shaw J, Paternoster B, Chandler N, Chitty LS (2020). Non-invasive prenatal diagnosis and screening for monogenic disorders. Eur J Obstet Gynecol Reprod Biol.

[11] Chiu RW, Chan KC, Gao Y, Lau VY, Zheng W, Leung TY (2008). Noninvasive prenatal diagnosis of fetal chromosomal aneuploidy by massively parallel genomic sequencing of DNA in maternal plasma. Proc Natl Acad Sci U S A.

[12] Lam KW, Jiang P, Liao GJ, Chan KC, Leung TY, Chiu RW (2012). Noninvasive prenatal diagnosis of monogenic diseases by targeted massively parallel sequencing of maternal plasma: application to beta-thalassemia. Clin Chem.

[13] Chan KC, Jiang P, Sun K, Cheng YK, Tong YK, Cheng SH (2016). Second generation noninvasive fetal genome analysis reveals de novo mutations, single-base parental inheritance, and preferred DNA ends. Proc Natl Acad Sci U S A.

[14] Lench N, Barrett A, Fielding S, McKay F, Hill M, Jenkins L (2013). The clinical implementation of non-invasive prenatal diagnosis for single-gene disorders: challenges and progress made. Prenat Diagn.

[15] Drury S, Mason S, McKay F, Lo K, Boustred C, Jenkins L (2016). Implementing non-invasive prenatal diagnosis (NIPD) in a national health service laboratory; from dominant to recessive disorders. Adv Exp Med Biol.

[16] Gruber A, Pacault M, El Khattabi LA, Vaucouleur N, Orhant L, Bienvenu T (2018). Non-invasive prenatal diagnosis of paternally inherited disorders from maternal plasma: detection of NF1 and CFTR mutations using droplet digital PCR. Clin Chem Lab Med.

[17] Guissart C, Dubucs C, Raynal C, Girardet A, Tran Mau Them F, Debant V (2017). Non-invasive prenatal diagnosis (NIPD) of cystic fibrosis: an optimized protocol using MEMO fluorescent PCR to detect the p.Phe508del mutation. J Cyst Fibros.

[18] Rabinowitz T, Shomron N (2020). Genome-wide noninvasive prenatal diagnosis of monogenic disorders: current and future trends. Comput Struct Biotechnol J.

[19] Li H, Du B, Jiang F, Guo Y, Wang Y, Zhang C (2019). Noninvasive prenatal diagnosis of β-thalassemia by relative haplotype dosage without analyzing proband. Mol Genet Genomic Med.

[20] Kitzman JO, Snyder MW, Ventura M, Lewis AP, Qiu R, Simmons LE (2012). Noninvasive whole-genome sequencing of a human fetus. Sci Transl Med.

[21] Lam K-WG, Jiang P, Liao GJW, Chan KCA, Leung TY, Chiu RWK (2012). Noninvasive prenatal diagnosis of monogenic diseases by targeted massively parallel sequencing of maternal plasma: application to β-thalassemia. Clin Chem.

[22] Fan HC, Gu W, Wang J, Blumenfeld YJ, El-Sayed YY, Quake SR (2012). Non-invasive prenatal measurement of the fetal genome. Nature.

[23] Rabinowitz T, Polsky A, Golan D, Danilevsky A, Shapira G, Raff C (2019). Bayesian-based noninvasive prenatal diagnosis of single-gene disorders. Genome Res.

[24] Lun FMF, Tsui NBY, Chan KCA, Leung TY, Lau TK, Charoenkwan P (2008). Noninvasive prenatal diagnosis of monogenic diseases by digital size selection and relative mutation dosage on DNA in maternal plasma. Proc Natl Acad Sci U S A.

[25] Hui WWI, Jiang P, Tong YK, Lee W-S, Cheng YKY, New MI (2017). Universal haplotype-based noninvasive prenatal testing for single gene diseases. Clin Chem.

[26] Zhang J, Li J, Saucier JB, Feng Y, Jiang Y, Sinson J (2019). Non-invasive prenatal sequencing for multiple Mendelian monogenic disorders using circulating cell-free fetal DNA. Nat Med.

[27] Lo YM, Lun FM, Chan KC, Tsui NB, Chong KC, Lau TK (2007). Digital PCR for the molecular detection of fetal chromosomal aneuploidy. Proc Natl Acad Sci U S A.

[28] Xu C, Wang T, Liu C, Li H, Chen X, Zhu H (2017). Noninvasive prenatal screening of fetal aneuploidy without massively parallel sequencing. Clin Chem.

[29] Mannucci PM, Tuddenham EG (2001). The hemophilias–from royal genes to gene therapy. N Engl J Med.

[30] Federici AB (2003). The factor VIII/von Willebrand factor complex: basic and clinical issues. Haematologica.

[31] Yee A, Kretz CA (2014). Von Willebrand factor: form for function. Semin Thromb Hemost.

[32] Kölker S, Garbade SF, Greenberg CR, Leonard JV, Saudubray J-M, Ribes A (2006). Natural history, outcome, and treatment efficacy in children and adults with glutaryl-CoA dehydrogenase deficiency. Pediatr Res.

[33] Gilmour DF (2015). Familial exudative vitreoretinopathy and related retinopathies. Eye (Lond).

[34] Li W, Wang Z, Sun Y, Wang Z, Bai J, Xing B (2019). A start codon mutation of the TSPAN12 gene in Chinese families causes clinical heterogeneous familial exudative vitreoretinopathy. Mol Genet Genomic Med.

[35] New MI, Tong YK, Yuen T, Jiang P, Pina C, Chan KCA (2014). Noninvasive prenatal diagnosis of congenital adrenal hyperplasia using cell-free fetal DNA in maternal plasma. J Clin Endocrinol Metab.

