# Supplementary figures and images for: Noninvasive fetal genotyping of single nucleotide variants and linkage analysis for prenatal diagnosis of monogenic disorders
Source: Hum Genomics. 2022 Jul 27;16:28. doi: 10.1186/s40246-022-00400-4 (PMC9327225; doi:10.1186/s40246-022-00400-4)

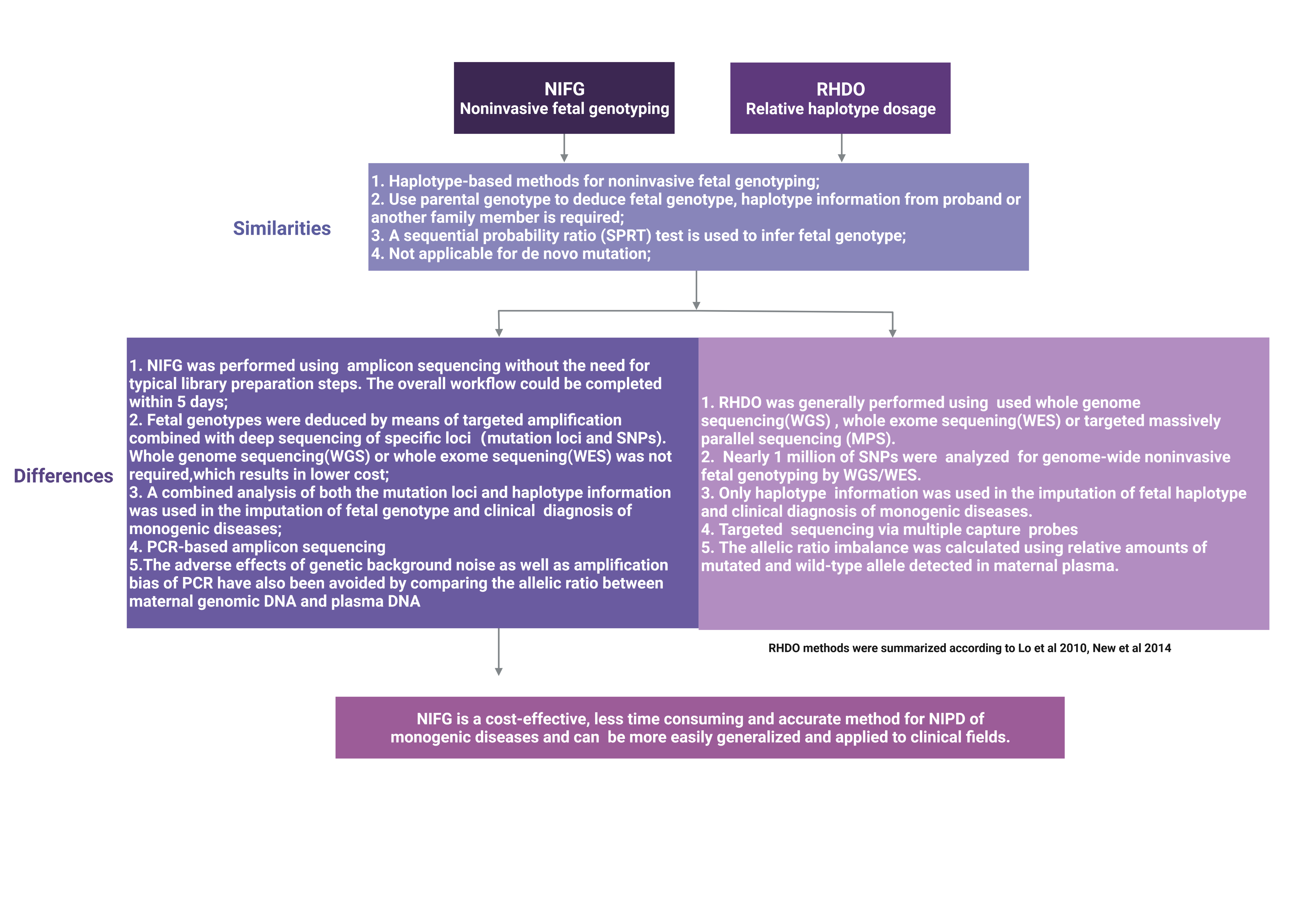

Supplement: Supplementary file 6 — Additional file 6. Figure S1: The similarities and differences between NIFG and RHDO methods. [file 40246_2022_400_MOESM6_ESM.jpeg]
